# The role of genetic factors in predisposition to squamous cell cancer of the head and neck

**DOI:** 10.1038/sj.bjc.6690138

**Published:** 1999-02

**Authors:** S Jefferies, R Eeles, D Goldgar, R A'Hern, J M Henk, M Gore, P Rhys-Evans, D Archer, K Bishop, A Murkin, E Solomon, S Hodgsun, M O'Connell, J Hibbert, D Easton, W Foulkes

**Affiliations:** Institute of Cancer Research and the Royal Marsden Hospital, Downs Road, Sutton, Surrey, SM2 5PT, UK; IARC, Lyon, France; Division of Medical Genetics, Department of Medicine, Montreal General Hospital, McGill University, Montreal, Canada; Guy's Hospital, St Thomas' Street, London, UK; IPH, Cambridge, UK

**Keywords:** genetic, squamous, head and neck cancer

## Abstract

© 1999 Cancer Research Campaign


British Journal of Cancer (1999) 79(5/6), 865–867
© 1999 Cancer Research Campaign
Article no. bjoc.1998.0138

### The role of genetic factors in predisposition to squamous cell cancer of the head and neck

**S Jefferies[1], R Eeles[1], D Goldgar[2], R A'Hern[1], JM Henk[1], M Gore[1], MPT Collaborators: P Rhys-Evans[1], D Archer[1], K Bishop[1], A Murkin[1], E Solomon[4], S Hodgsun[4], M O'Connell[4], J Hibbert[4], D Easton[5] and W Foulkes[3]**

[1]Institute of Cancer Research and the Royal Marsden Hospital, Downs Road, Sutton, Surrey SM2 5PT, UK; [2]IARC, Lyon, France; [3]Division of Medical Genetics, Department of Medicine, Montreal General Hospital, McGill University, Montreal, Canada; [4]Guy's Hospital, St Thomas' Street, London, UK; [5]IPH, Cambridge, UK

**Keywords:** genetic; squamous; head and neck cancer

Squamous cell carcinoma of the head and neck (SCCHN) is the sixth commonest human malignancy with an estimate of nearly 900 000 new cases occurring worldwide in 1995 (Parkin et al, 1993). In this review, head and neck cancer is defined as squamous cell carcinoma occurring in the upper aerodigestive tract, superior to the clavicles, excluding skin cancer and nasopharyngeal carcinoma. The incidence of head and neck cancer is increasing in developing countries (Macfarlane et al, 1994). In England and Wales, the overall incidence is stable at 5000 cases/year ($14/10^5$ of the male and $5.5/10^5$ of the female population), but within the head and neck sites, tumours of the larynx and oral cavity are increasing.

Optimal management of SCCHN is by a multidisciplinary approach combining surgery, radiotherapy and more recently chemotherapy. The presenting tumour site and nodal status have independent implications for both treatment and prognosis. Despite therapeutic changes the overall survival rate from SCCHN remains poor (Vokes et al, 1993). A component contributing to the poor overall survival, particularly in patients initially presenting with early tumours of the larynx, oral cavity and oropharynx, is the 10–30% chance of developing a second malignancy or multiple primary tumour (MPT) of the aerodigestive tract or elsewhere within 5 years (Day, 1992; Jovanic et al, 1994).

There are various criteria for multiple primary tumour development. A classification described by Hong provides strict criteria to exclude recurrence of SCCHN (Hong et al, 1990). This includes:

1. A MPT must occur more than 3 years from the original diagnosis or be separated by greater than 2 cm of entirely normal epithelium.
2. For a MPT of different histology these criteria do not apply.
3. If occurring in the lung, a MPT must be solitary and histologically distinct if less than 3 years after the first tumour.
4. A MPT is not limited to the upper aerodigestive tract.

The increasing relevance of SCCHN, together with failure to improve survival rates, emphasizes the need for better understanding of SCCHN epidemiology. Greater understanding of the disease process may then allow targeted primary prevention of SCCHN and secondary prevention for those at risk of MPT with subsequent improvement in both quality and duration of life.

Slaughter et al (1953) proposed the concept of field cancerization, which suggests that the whole of the upper aerodigestive tract is exposed to the damaging effects of environmental agents and therefore is at risk of multiple tumour development. The field cancerization hypothesis predicts that second or multiple primary tumours occur as independent genetic events. Environmental agents are undoubtedly very important when considering risks of developing SCCHN. There is an abundance of information on tobacco and alcohol. These agents increase risk in a dose-dependent and independent manner. In non-drinkers, the effect of smoking is to increase relative risk (RR) of developing SCCHN, the increase varies between 2 and 20-fold. In some sites, larynx, oral and pharyngeal cancer, the combined effect of alcohol and tobacco can be multiplicative for cancer risk (RR: 2–140) (Maier et al, 1992). Areca nut exposure, alone or as a component of pan/betel quid, is associated with SCCHN. Tobacco and alcohol in combination with areca nut increase the risk of developing SCCHN (van Wyck et al, 1993; Patel et al, 1994). A smaller increase in relative risk has also been demonstrated from exposure to asbestos (RR: 1.3) (Muscat and Wynder, 1992), coal products (Maier et al, 1991), viral infections (Larson et al, 1991; Fouret et al, 1995) and long-term consumption of nitrosamine-containing food (Rogers et al, 1995). Risk factors differ for different sites and, therefore, when conducting epidemiological studies this must be taken into account.

Despite the clearly defined evidence of the role of environmental agents in the development of SCCHN there is also a possibility that inherited susceptibility may be be important.

### EVIDENCE SUGGESTING A GENETIC COMPONENT IN SCCHN

### Familial aggregates of cancer and increased cancer risks amongst relatives

The occurrence of familial aggregations of cancer suggests existence of genetic predisposing factors. Families occur that exhibit a high number of cases of cancer, which also tend to occur at younger ages. A familial cluster of oral squamous cell cancer has been reported which also demonstrated cases occurring at younger ages (Ankathil et al, 1996). A study addressing the prevalence of

*Received 14 April 1998*
*Revised 3 August 1998*
*Accepted 19 October 1998*

*Correspondence to:* S Jefferies

**Table 1** Relative risk of SCCHN and RUDT in first-degree relatives of cases of SCCHN

| Authors | Copper et al, 1995 | Foulkes et al, 1995 | Goldstein et al, 1995 | Foulkes et al, 1996 |
|---|---|---|---|---|
| Location | The Netherlands | Brazil | USA | Quebec, Canada |
| No. of cases | 105 | 754 | 1065 | 242 |
| Type | Consecutive | Incident | Incident and prevalent | Incident and prevalent |
| Source | ENT clinic | Hospital cases | Cancer registry | Head and neck clinic |
| No. of controls | 105 | 1509 | 1182 | 156 |
| Type | Spouse | Hospital | Population register | Spouse |
| First-degree relatives: | | | | |
| Cases | 617 | – | – | 1429 |
| Controls | 618 | – | – | 934 |
| Relative details: | | | | |
| Cancer | Not verified | Not verified | Not verified | Not verified |
| Smoking | Collected | Collected | Collected | Collected |
| Alcohol | Not collected | Not collected | Not collected | Collected |
| RR of SCCHN in relatives of SCCHN cases (95% CI) | RR = 3.4 (0.8–13.7) | RR = 3.5 (1.97–6.76) | – | RR = 3.79 (1.11–13.00) |
| Subgroup analysis (95% CI) | RUDT RR = 3.5 (1.90–6.40) Sibling RR = 14 (3.10–69.10) | Sibling RR = 8.57 (2.72–27.04) | Pharynx RR = 1.7 (1.10–2.80) Oral RR = 1.2 (0.80–1.70) | Pharynx RR = 4.24 (1.02–17.6) Oral RR = 3.82 (1.08–13.50) Larynx RR = 1.65 (0.35–7.61) |

RUDT—respiratory and upper digestive tract; RR—relative risk; SCCHN—squamous cell carcinoma of the head and neck; CI—confidence interval.

oral cancer in an Israeli Jewish population found that, after controlling for smoking and alcohol usage, an Ashkenazi group had at least twice the risk of developing oral cancer compared with other Jewish groups (Gorsky et al, 1994).

Increased risk of cancer is noted amongst relatives of patients with SCCHN. Four recent epidemiological studies in SCCHN have addressed the issue of familial factors in detail; these are summarized in Table 1. Copper et al (1995) found that first-degree relatives of patients with head and neck cancer had an increased incidence of upper aerodigestive tract tumours (RR: 3.5). A higher rate of upper aerodigestive tract cancer was observed amongst siblings (RR: 14.6). Foulkes et al (1995) performed a study in Southern Brazil which demonstrated that relative risk for developing SCCHN, if a first-degree relative had SCCHN, was 3.5. In the same study, the relative risk of SCCHN if a sibling had developed the disease was 8.6. In a historical cohort study by the same author (Foulkes et al, 1996) in Quebec, a relative risk of 3.79 was seen for SCCHN in association with a family history of SCCHN. All three studies have shown a similar magnitude of relative risk. In contrast to this, a study by Goldstein et al (1994) found only a slight increase in relative risk of SCCHN within families of oral and pharyngeal cases.

There are a number of factors relating to these studies that need to be considered. In each of these studies cases of cancer amongst relatives were not confirmed. Attempts to collect information on relatives' alcohol consumption proved to be unreliable in all of the studies. Analysis of the first-degree relative characteristics in the studies that used spouse controls show that demographic factors and smoking were well matched. Of note in the Goldstein study is that smoking details were not collected for relatives and matching was by racial group.

What can be concluded from these studies? The study by Goldstein suggested that, at most, there was a weak familial link between oral/pharyngeal cancer. In this study, black racial groups with a brother with cancer had an increased risk of oral cancer (odds ratio (OR): 7.4; confidence interval (CI): 1.8–31). This effect was not seen in whites. The studies by Copper (1995) and Foulkes et al (1996) (Quebec) found that familial factors may be stronger for oral and pharyngeal than for laryngeal cancer. The Brazilian study did not show any differences by tumour site for familial risk. It therefore appears that there may be a familial susceptibility to SCCHN, which is not entirely accounted for by a shared environment.

**Table 2** Relative risk of SCCHN and RUDT in first-degree relatives of MPT cases

| Authors | Foulkes et al, 1996 | Bongers et al, 1996 |
|---|---|---|
| No. of cases of single SCCHN | 222 | 100 |
| No. of cases of MPT | 20 | 97 |
| Hong criteria | Yes | Yes |
| Endpoints | RR of SCCHN in first-degree relatives | Incidence of SCCHN and RUDT in first-degree relatives (%) |
| Single SCCHN | RR = 3.79 | 2.5% |
| MPT | RR = 7.89 | 8.9% |
| *P*-value | $P = 0.009$ | $P < 0.00001$ |

RUDT—respiratory and upper digestive tract; RR—relative risk; MPT—multiple primary tumours; SCCHN—squamous cell carcinoma in the head and neck.

### Multiple primary tumours

There is evidence from other sites that genetically predisposed individuals tend to develop multiple cancers (e.g. bilateral breast cancer is more likely to be familial) (Ottman et al, 1983). A study comparing family history of upper aerodigestive tract tumours amongst 100 patients with second primary tumours and 100 patients

 *© Cancer Research Campaign 1999*

with single-site SCCHN (prevalent cases at least 6 years from diagnosis) was performed by Bongers et al (1996) (Table 2). This demonstrated that, if one or more relatives had an upper aerodigestive tract tumour, this was a risk factor for MPT (OR: 3.8; CI: 2.0–7.6) (Bongers et al, 1996). Foulkes et al demonstrated that the familial risk for SCCHN in relatives of cases with multiple primary SCCHN was greater than for relatives with a single primary SCCHN (RR: 7.89 vs. 3.53) and statistically significantly greater than for controls. However, this was based upon only 26 cases with MPT (Foulkesl, 1996). Taken together with the markedly increased RR of cancer in relatives of individuals with MPT, the occurrence of MPT increases the likelihood of an underlying genetic basis for SCCHN, at least within the individuals with MPT.

### Rare cancer syndromes

A number of rare syndromes occur that are clearly genetically determined where there is a marked increased cancer risk amongst gene carriers. Cancer tends to occur at younger ages and occurs in the absence of carcinogen exposure. SCCHN is occasionally featured in several inherited cancer syndromes, including families with hereditary non-polyposis colo-rectal cancer, Fanconi's anaemia, Bloom's syndrome, Xeroderma Pigmentosum and Li-Fraumeni syndrome (Trizna and Schantz, 1992). *P*16 germline mutations have been seen in individuals from families with melanoma, pancreatic tumours and SCCHN (Yarbrough et al, 1996; Sun et al, 1997).

### CONCLUSION AND FUTURE CLINICAL IMPLICATIONS

The aetiology of SCCHN has been clearly defined in terms of the environmental factors that predispose to this condition but until recently little attention has been paid to the possible role of genetic factors in SCCHN. Three recent case-control studies indicate that family history of SCCHN is a significant risk factor for SCCHN particularly if MPT has occurred; however, only very small numbers of patients with MPT have been studied. Despite the influence of environmental factors on the development of SCCHN, it is now important to link epidemiological and clinical data with molecular markers from tumour specimens in prospective clinical studies. This information may have important preventative and therapeutic implications. We have commenced a prospective collaborative study, supported by the Cancer Research Campaign, to address exactly these issues.*

### REFERENCES

Ankathil R, Mathew A and Joseph F (1996) Is oral cancer susceptibility inherited? *Eur J Oral Oncol* **32B**: 63–67

Bongers V, Braakhuis B and Tobi H (1996) The relation between cancer incidence among relatives and the occurrence of multiple primary carcinomas following head and neck cancer. *Cancer Epidemiol Biomarker Prev* **5/8**: 595–598

Copper M, Jovanic A and Nauta J (1995) Role of genetic factors in the aetiology of squamous cell carcinoma of the head and neck. *Arch Otolaryngol Head Neck Surg* **121**: 157–160

Day GL and Blot WJ (1992) Second primary tumours in patients with oral cancer. *Cancer* **70**: 14–19

Foulkes WD, Brunet J-S and Kowalski LP (1995) Family history is a risk factor for squamous cell carcinoma of the head and neck in Brazil: a case-control study. *Int J Cancer* **63**: 769–773

Foulkes WD, Brunet J-S, Sieh W, Black MJ, Shenouda G and Narod SA (1996) Familial risk of squamous cell carcinoma: a retrospective case-control study. *Br Med J* **313**: 716–721

Fouret P, Martin F and Flahault A (1995) HPV infection in the malignant and premalignant head and neck epithelium. *Diagn Mol Pathol* **4**: 122–127

Goldstein A, Blot W and Greenberg R (1994) Familial risk in oral and pharyngeal cancer. *Eur J Cancer B Oral Oncol* **30B**: 319–322

Gorsky M, Littner M and Sukman Y (1994) The prevalence of oral cancer in relation to the ethnic origin of the Jewish population. *Oral Surg Oral Med Oral Pathol* **78(3)**: 408–411

Hong WK, Lippman SM, Itri LM, Karp DD, Lee JS and Byers RM (1990) Prevention of second primary tumours with isoretinoin squamous-cell carcinoma of the head and neck. *N Engl J Med* **323**: 795–801

Jovanic A, Ignaz GH and Pieter J (1994) Second respiratory and upper digestive tract cancer following oral squamous cell carcinoma. *Oral Oncol Eur J Cancer* **30B**: 225–229

Larrson PA, Edstrom S and Westin T (1991) Reactivity against HSV in patients with head and neck cancer. *Int J Cancer* **49**: 14–18

Maier H, Dietz A and Gewelke U (1991) Occupational exposure to hazardous substances and risk of cancer of the oral cavity, hypopharynx and larynx. *Laryngol Rhinol Otol* **70**: 93–98

Maier H, Dietz A and Gewelke U (1992) Tobacco, alcohol and the risk of head and neck cancer. *Clin Invest* **70**: 320–327

Macfarlane G, Boyle P and Evstifeeva T (1994) Rising trends of oral cancer mortality among males worldwide: the return of an old public health problem. *Cancer Causes Control* **5**: 259–265

Muscat J and Wynder E (1992) Tobacco, alcohol, asbestos and occupational risk factors for laryngeal cancers. *Cancer* **69**: 2244–2251

Ottman R, Pike MC, King M-Cand Henderson BE (1983) Practical guide for estimating risk for familial breast cancer. *Lancet* **ii**: 556–558

Parkin DM, Pisani P and Ferlay J (1993) Estimates of the world-wide incidence of eighteen major cancers in 1995. *Int J Cancer* **54**: 594–606

Patel R, Trivedi A and Jaju R (1994) Ethanol potentiates the clastogenicity of pan masala. *Carcinogenesis* **15**: 2017–2021

Rogers M, Vaughn T and Davis S (1995) Consumption of nitrite nitrate and nitrodimethylamine and the risk of upper aerodigestive tract cancer. *Cancer Epidemiol Biomark Prev* **4**: 29–36

Slaughter DP, Southwick HW and Smejkal W (1953) Field cancerisation in oral stratified squamous epithelium. *Cancer* **6**: 963–968

Sun S, Pollock P and Liu L (1997) *CDKN2A* mutation in a non-FAMM kindred with cancers at multiple sites results in a functionally abnormal protein. *Int J Cancer* **73**: 531–536

Trizna Z and Schantz SP (1992) Hereditary and environmental factors associated with risk and progression of head and neck cancer. *Otolaryngol Clin North Am* **25**: 1089–1103

Van Wyck C, Stander I and Padayachee A (1993) The areca nut and oral squamous cell carcinoma in South African Indians. *S Afr Med J* **83**: 425–429

Vokes EE, Weichselbaum RR, Lippman SM and Hong WK (1993) *N Engl J Med* **328**: 184–194

Yarbrough W, Apelikova O and Pei H (1996) Familial tumour syndrome associated with a germline non-functional p16 allele. *J Natl Cancer Inst* **88**: 1489–1490

*For further information about this study please contact Dr S J Jefferies, Cancer Genetics Unit, Royal Marsden Hospital, Downs Rd, Sutton, Surrey SM2 5PT, UK. E-mail: sarahj@icr.ac.uk

© Cancer Research Campaign 1999
